# Meetings of Societies

**Published:** 1919-12

**Authors:** 


### MEETINGS OF SOCIETIES.

### Bristol Medico-Chirurgical Society.

*Annual Meeting, October 8th,* 1919.

After a vote of thanks to the retiring President, Mr. R. G. Poole Lansdown, had been passed, Dr. I. Walker Hall took the chair and gave an introductory address (*see* p. 105). Later a cordial vote of thanks was given to Dr. Walker Hall for his able and interesting address.

The Secretary, Mr. A. J. Wright, and the Editorial Secretary, Dr. J. M. Fortescue-Brickdale, read their Annual Reports, Dr. G. Parker read the Library Report in the absence of the Hon. Librarian, Mr. C. K. Rudge, which was as follows :—

The Library of the Bristol Medico-Chirurgical Society now contains 13,279 bound volumes. During the year 175 volumes have been added—of this number 77 were received through the *Journal.*

Owing to the excessive cost, it has not been possible to bind as many journals as formerly, and there are now some 220 unbound volumes.

During the year 53 duplicate books were disposed of, and readers have borrowed 342 volumes.

The Library is in regular receipt of 88 periodicals. An effort to replace the periodicals lost owing to the war was made in May, and members were asked to be responsible for certain periodicals.

The Bristol Medical Library, to which members of the Society have access, now contains 24,209 volumes, and is in receipt of 147 current periodicals.

The strength of the various libraries is as follows :—

BOOKS.

| | |
|---|---|
| Medico-Chirurgical Society .. .. | 13,279 |
| University Medical Library .. .. | 9,211 |
| Other sources .. .. .. .. | 1,719 |
| | 24,209 |

CURRENT PERIODICALS.

| | |
|---|---|
| Medico-Chirurgical Society .. .. | 88 |
| University Medical Library .. .. | 59 |
| | 147 |

The following are the officers of the Society for the Session 1919–1920 : President—I. Walker Hall, M.D.Vict. President-Elect—L. E. V. Every-Clayton, M.D. Lond. Committee (*ex officio*)—R. G. P. Lansdown, M.D. Durh. (retiring President), P. Watson Williams, M.D. Lond. (Editor of *Journal*) C. K. Rudge, M.R.C.S. (Hon. Librarian) ; J. M. Fortescue-Brickdale, M.A., M.D. Oxon. (Editorial Secretary) ; A. J. Wright, M.B. Lond. (Hon. Secretary). (*Elected*)—R. Shingleton Smith, M.D. Lond. ; G. Parker, M.D. Cantab. ; J. Lacy Firth, M.S. Lond. ; J. O. Symes, M.D. Lond. ; T. Carwardine, M.S. Lond. ; D. C. Rayner, F.R.C.S. ; John Wallace, M.B., C.M. Edin. ; J. A. Nixon, C.M.G., M.D. Cantab. Library Committee—C. K. Rudge, M.R.C.S. ; W. A. Smith, M.D. ; George Parker, M.D.

### RECEIPT AND EXPENDITURE ACCOUNT OF THE BRISTOL MEDICO-CHIRURGICAL SOCIETY, SESSION 1918-19.

| RECEIPTS. | £ | s. | d. | EXPENDITURE. | £ | s. | d. |
|---|---|---|---|---|---|---|---|
| Balance from 1918 .. | 178 | 10 | 10 | University of Bristol (Rent) .. .. .. | 20 | 0 | 0 |
| Members' Subscriptions | 124 | 19 | 0 | Librarian, Honorarium | 15 | 16 | 0 |
| Interest on Deposit .. | 3 | 6 | 9 | Library Grant .. .. | 25 | 0 | 0 |
| | | | | *Journal* Grant .. .. | 38 | 15 | 0 |
| | | | | Audit Fee .. .. .. | 2 | 2 | 0 |
| | | | | Subscriptions refunded.. | 8 | 8 | 0 |
| | | | | Miscellaneous Expenses | 9 | 13 | 5 |
| | | | | Balance in Bank .. .. | 187 | 2 | 2 |
| | £306 | 16 | 7 | | £306 | 16 | 7 |

Audited and found correct,

CURTIS, JENKINS & CO.,
*Chartered Accountants.*

*3rd October,* 1919.

---

*November 12th,* 1919.

I. Walker Hall, M.D., President, in the Chair.

Dr. E. C. Williams showed the following cases :—

1. **Pseudo-Hypertrophic Muscular Atrophy.**
2. **Cerebral Diplegia.**
3. **Ataxia.**

Mr. Hey Groves showed cases of and read a paper on **"Internal Derangement of the Knee Joint."**

Dr. Scott Williamson read a paper on **"The Clinical Value of Blood and Urine Analysis."**

### Proposed change of Rule 7.

The President read the proposition of the Committee that Rule 7 shall be altered to read as follows :—

The Subscription of the Society shall be One and a Half Guineas per annum, payable in advance ; this sum shall include the subscription to the *Journal* and the use of the Library, with the privileges appertaining to the circulation of Library Books.

The following Notice to Members had been previously circulated :—

The Committee has had under consideration the ways and means of maintaining the objects of the Society during the period of increased cost of materials, etc.

The Society has hitherto published a quarterly journal, consisting of approximately 96 pages.

In 1914 each number was produced for £36.

In 1919 each issue will cost £62.

In 1914 the distribution cost £3 per number.

In 1919 the distribution will cost £6 per number.

The Committee recommends the Society to provide for the continuance of the *Journal,* to maintain thereby the value of the Society to those members who are not able to attend the meetings, and to continue the support the Society has given to Bristol as a teaching and scientific centre.

The Committee therefore proposes the following resolutions :

"That the Subscription to the Bristol Medico-Chirurgical Society be increased to One and a Half Guineas per annum, and that the Subscription to the *Journal* be raised to 10/6 per annum."

"That owing to the special circumstances, members shall be invited to pay the increased subscription from October, 1919."

These resolutions were agreed to unanimously.

J. Lacy Firth.
A. J. Wright, *Hon. Sec.*

